# Mathematical Modeling of Hepatitis C Prevalence Reduction with Antiviral Treatment Scale-Up in Persons Who Inject Drugs in Metropolitan Chicago

**DOI:** 10.1371/journal.pone.0135901

**Published:** 2015-08-21

**Authors:** Desarae Echevarria, Alexander Gutfraind, Basmattee Boodram, Marian Major, Sara Del Valle, Scott J Cotler, Harel Dahari

**Affiliations:** 1 The Program for Experimental & Theoretical Modeling, Division of Hepatology, Department of Medicine, Loyola University Chicago, Medical Center, Maywood, Illinois, United States of America; 2 Division of Epidemiology and Biostatistics, School of Public Health, University of Illinois at Chicago, Chicago, Illinois, United States of America; 3 Division of Viral Products, Center for Biologics Evaluation and Research, Food and Drug Administration, Silver Spring, Maryland, United States of America; 4 Energy and Infrastructure Analysis Group, Los Alamos National Laboratory, Los Alamos, New Mexico, United States of America; 5 Theoretical and Biophysics Group, Los Alamos National Laboratory, Los Alamos, New Mexico, United States of America; University of Cincinnati College of Medicine, UNITED STATES

## Abstract

**Background/Aim:**

New direct-acting antivirals (DAAs) provide an opportunity to combat hepatitis C virus (HCV) infection in persons who inject drugs (PWID). Here we use a mathematical model to predict the impact of a DAA-treatment scale-up on HCV prevalence among PWID and the estimated cost in metropolitan Chicago.

**Methods:**

To estimate the HCV antibody and HCV-RNA (chronic infection) prevalence among the metropolitan Chicago PWID population, we used empirical data from three large epidemiological studies. Cost of DAAs is assumed $50,000 per person.

**Results:**

Approximately 32,000 PWID reside in metropolitan Chicago with an estimated HCV-RNA prevalence of 47% or 15,040 cases. Approximately 22,000 PWID (69% of the total PWID population) attend harm reduction (HR) programs, such as syringe exchange programs, and have an estimated HCV-RNA prevalence of 30%. There are about 11,000 young PWID (<30 years old) with an estimated HCV-RNA prevalence of 10% (PWID in these two subpopulations overlap). The model suggests that the following treatment scale-up is needed to reduce the baseline HCV-RNA prevalence by one-half over 10 years of treatment [cost per year, min-max in millions]: 35 per 1,000 [$50-$77] in the overall PWID population, 19 per 1,000 [$20-$26] for persons in HR programs, and 5 per 1,000 [$3-$4] for young PWID.

**Conclusions:**

Treatment scale-up could dramatically reduce the prevalence of chronic HCV infection among PWID in Chicago, who are the main reservoir for on-going HCV transmission. Focusing treatment on PWID attending HR programs and/or young PWID could have a significant impact on HCV prevalence in these subpopulations at an attainable cost.

## Introduction

The global prevalence of hepatitis C (HCV) antibody (Ab) is about 180 million, with approximately 500,000 HCV-related deaths per year [1, 2]. In the United States (U.S.), an estimated 4.1 million individuals are HCV-Ab positive [3] (~3.2 million are chronically infected), with an additional 30,000 new (incident) cases of HCV infection occurring each year [4]. The primary mode of HCV transmission in developed countries is injection drug use (IDU) and it is estimated that 60% of all HCV infections are attributable to sharing syringes and other drug paraphernalia [5]. There is no vaccine for HCV and less than 1% of HCV-infected persons who inject drugs (PWID) are treated annually with interferon-based antiviral medications [6, 7]. The majority of PWID in the U.S. are infected with HCV genotype-1, which was the most difficult to treat genotype with interferon [8]. This has changed with the advent of direct-acting antivirals (DAAs) [9] that provide interferon-free, all-oral treatment yielding cure rates exceeding 90% for genotype 1. However, there are several barriers restricting access to treatment for PWID including cost [10], Medicaid restrictions on sofosbuvir—containing regimens based on liver disease stage, and poor treatment readiness due to a drug-use lifestyle that may lead to re-exposure after treatment [11].

Immunity to HCV infection, either before or after treatment, is also important to consider. The presence of some form of natural immunity to chronic infection in patients who spontaneously clear HCV is well documented [12, 13]. This natural immunity among some PWID who spontaneously clear the virus could have an impact on subsequently treatment scale-up in this population. However, it is less clear if a similar type of protection from secondary infections exists following treatment. Functional CD4+ and CD8+ T-cell responses have been shown in patients following early treatment of acute phase HCV infections, while these are absent or weak in patients successfully treated during the chronic phase [14, 15]. Thus, the stage at which an infection is treated could also affect the long-term impact of drug intervention within a PWID population and treatment scale-up.

Given the illicit nature of IDU in the U.S., most of the PWID population is hidden, making it problematic to rely on empirical data to assess treatment and intervention cost-effectiveness in this population. Mathematical modeling provides an alternative approach to understanding HCV spread (reviewed in [16]). International studies by Martin et al. projected that HCV treatment may be cost effective in achieving desirable reduction in HCV prevalence in some PWID populations [17–19].

The well-characterized HCV epidemic in Chicago PWID [20–22] represents a unique opportunity to examine DAA-treatment scale-up in the U.S. using a mathematical modeling approach. We applied the model developed by Martin et al. [17] to the Chicago PWID population to assess the impact of DAA treatment scale-up on HCV-viral load (RNA) prevalence among PWID populations, accounting for the varied prevalence observed in sub-populations from empirical studies on Chicago PWID [21–25]. We updated some of the parameter values used by Martin et al. [17] based on recent clinical data showing increased cure rates (or sustained virological response (SVR) rates) and shorter treatment duration with the latest antivirals [9, 26], and studies consistently indicating a higher probability of acquiring specific immunity after spontaneous clearance both in humans and chimpanzees [12, 13, 27–31]. Our model reports, for the first time, the potential public health impact and estimated cost of new DAA therapy scale-up for HCV among PWID populations in a large metropolitan area in the U.S.

## Methods

### HCV prevalence estimation in metropolitan Chicago

To estimate the prevalence of HCV antibody and HCV-RNA among the 32,000 PWID residing in metropolitan Chicago [20], we relied on empirical data collected on the metropolitan Chicago PWID population. These include: (i) the Chicago site from the CDC-sponsored 2009 National HIV Behavioral Surveillance System (NHBS) [22, 23, 32], (ii) the Third Collaborative Injection Drug Users (CIDUS III, 2002–2004) study [21, 24] and (iii) the Early Natural History of HCV Infection Among Injection Drug Users (NATHCV, 2002–2006) study [25]. The Chicago site of NHBS recruited 545 PWID using respondent-driven sampling (RDS), which has been shown to be effective in recruiting hard to reach populations like PWID [33]. The NHBS population was 43% non-Hispanic (NH) Black, 33% Hispanic (all races), 24% NH-White, and had an overall median age of 44 years old. The overall HCV-Ab prevalence was 59%, with NH-Blacks having the highest prevalence (66%), followed by Hispanics (64%), and NH-White (38%) [22, 32]. Since 2003, to monitor behaviors associated with HIV/HCV risk among PWID, the CDC's NHBS conducts interviews and HIV/HCV testing in selected cities across the U.S. using highly standardized, stringent methodologies for recruitment PWID using RDS. The values we report are consistent across multiple studies of the Chicago PWID population. In particular, the 2009 and 2012 NHBS reported Ab prevalence of 59% and 54%, respectively. Approximately 22,000 PWID (69% of the total PWID population) attend harm reduction (HR) programs, such as syringe exchange programs, and have an estimated HCV-RNA prevalence of 30%. There are about 11,000 young PWID (<30 years old) with an estimated HCV-RNA prevalence of 10% (PWID in these two subpopulations overlap). CIDUS III [21, 24] was a multi-city randomized-controlled HIV intervention trial that recruited young (15–30 years old) PWID using RDS. Chicago recruited 796 PWID who were predominantly NH-White (75%), with Hispanics (18%) being the next largest group. The overall Chicago HCV Ab prevalence among young PWID was 14% [24]. The Early Natural History of HCV Infection Among Injection Drug Users NATHCV (2002–2006) was a longitudinal study that followed 125 HCV Ab-positive young PWID (aged 18–35) [25], most of whom were CIDUS III participants who tested HCV-Ab positive at the baseline visit. Thirty-four percent of NATHCV participants had evidence of cleared HCV infection (i.e., only 66% were HCV-RNA positive) at the baseline assessment. Given the cost [10] and highly restricted access to DAAs by PWID in Illinois [34], antiviral treatment is estimated to be as low as 1 per 1000 PWID treated annually in Chicago, and therefore, has a negligible effect on HCV prevalence estimates. All study procedures for the CIDUS III and NATHCV data were approved by the Institutional Review Board of the University of Illinois at Chicago and the use of the NHBS data was approved through a data agreement with the Chicago Department of Public Health.

### Mathematical Modeling

**Fig 1:**
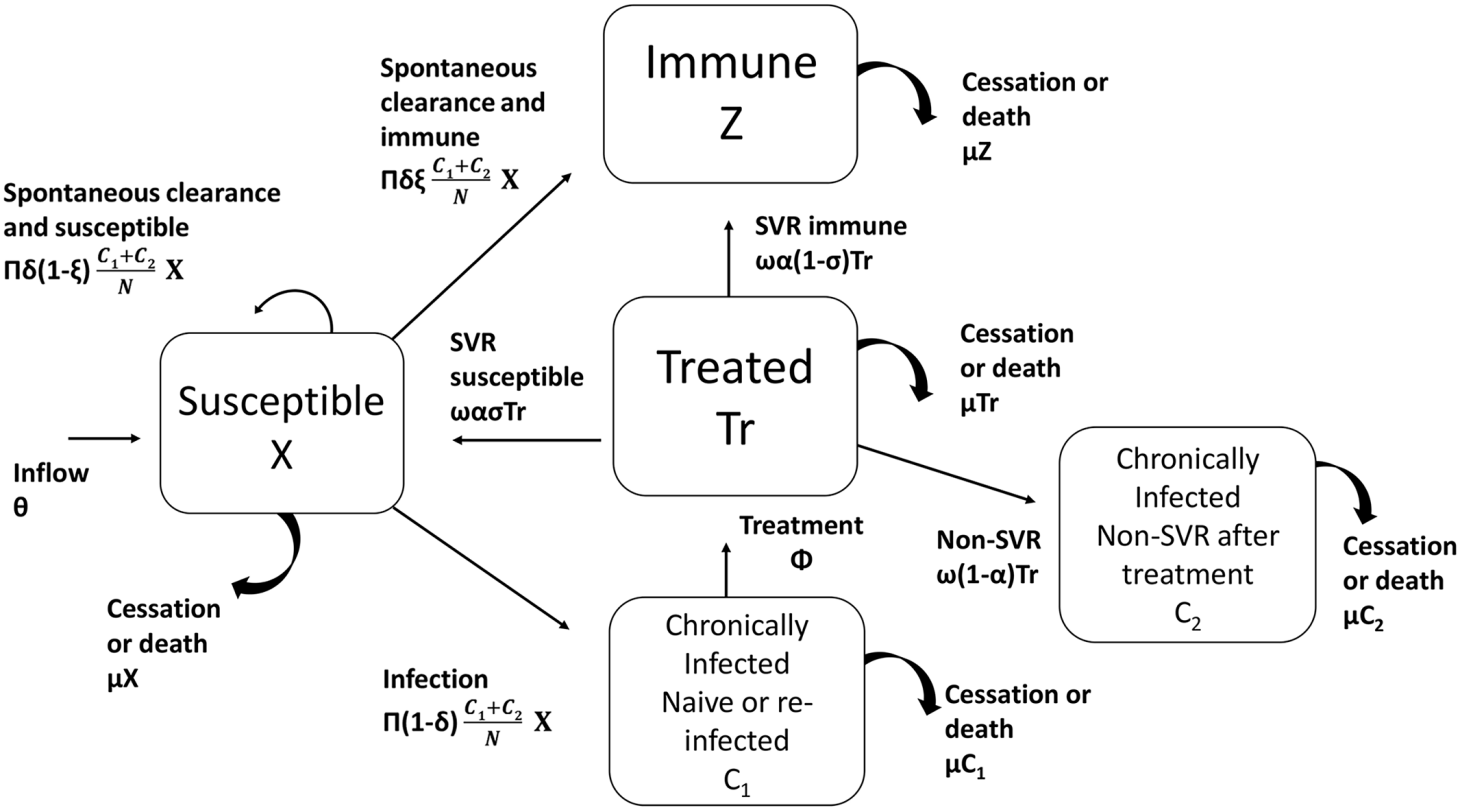
Schematic description of Martin et al. [17] mathematical model. N represents the total PWID population (X+Tr+Z+C_1_+C_2_). Model parameters are described in Table 1.

The model developed by Martin et al. [17] was applied in this study (Fig 1 and S1 Eq). The model describes five possible states to which a given PWID may belong during the simulation period: (i) X = susceptible to HCV infection including naïve, a small fraction (~20%) of those who cleared HCV-infection spontaneously and approximately all PWID who were cured after antiviral treatment (i.e., SVR) and did not develop immunity (or resistance to re-infection), (ii) C_1_ = chronically infected PWID (i.e., detectable HCV-RNA in persons who are treatment naïve or reinfected following antiviral treatment) (iii) C_2_ = chronically infected individuals who have failed treatment (non SVR), (iv) Tr = on antiviral treatment, and (v) Z = about 80% of those who acquired immunity after clearing the virus spontaneously and are not susceptible to developing chronic infection after re-infection. A detailed explanation of model equations, parameters and their values is provided in Table 1, S1 Eq, and in the following section.

**Table 1:** Model parameters.

Model parameter definition	Formula	Value [Range]	Units	Source
**Population Dynamics**				
**Average new injector rate** *	θ	θ = 85 [50–200]	Per 1000 PWID annually	[17]
**Average PWID leaving rate** * **(cessation or death)**	μ	μ = 0.085 [0.05–0.2]	Per year	[17]
**Infection Dynamics**				
**Average HCV infection rate per year**	π	Based on H_RNA_P π ∈[0–0.95]	Per year	**
**Average proportion of infections that spontaneously clear the infection**	δ	δ = 0.26 [0.22–0.34]	Per PWID	[17, 25]
**Average proportion of spontaneously cleared infections resulting in immunity**	ξ	ξ = 0.8 [0–0.8]	Per PWID	[27–30]
**Treatment Dynamics**				
**Average treatment rate**	Φ	Φ = 0 [5–40]	Per 1000 PWID annually	[17]
**Average treatment duration**	1/ω	ω = 0.23	Year	[9]
**Average proportion of cured infections due to treatment resulting in immunity**	1-σ	σ = 1 [0.75–1.0]	Per PWID	[17]
**Average proportion of cured infections with SVR**	α	α = 0.9	Per PWID	[9]

* In sensitivity analysis we keep the relationship θ = 1000*μ to maintain a steady population size.

** Calibrated for each population to produce the empirically observed prevalence rate before initiation of antiviral scale-up.

### Model parameters and new assumptions

The model parameters and their values detailed by Martin et al. [17] (Table 1), were used with some modifications. First, Martin et al. assumed that 26% of PWID with primary HCV infection spontaneously cleared the virus with no or very limited acquired specific immunity (i.e., parameter ξ = 0, ranged [0–0.25]). Based on Osburn et al [12] findings of 83% protective immunity in humans (which is in agreement with available human data [13, 27, 28, 31, 35] and data from 99 chimpanzees [29, 30]), we assume that the rate of acquired immunity is much higher (ξ = 0.8, range [0–0.8]), i.e., 80% of those that spontaneously clear HCV are protected from chronic infection upon secondary exposure. Based on recent clinical trials with new DAAs [9, 10, 26, 36], we also assume high SVR rates (90%) and shorter treatment durations (6 or 12 weeks), although in some cases longer duration (up to 24 weeks) of therapy may be needed to achieve cure. We also assume different prevalences based on the empirical data collected on the metropolitan Chicago PWID population. Three main prevalences were reproduced—total PWID population, HR population, and the young PWID—in the model, by varying the infection rate, π. Martin et al. [17] used δ = 0.26, but in a previous study of a young PWID population [25], we found that 34% of HCV Ab positive young PWID cleared the virus. Therefore, δ = 0.26 was used for the total PWID population and HR population, and δ = 0.34 was used for the young PWID.

## Results

### Higher immunity leads to lower baseline HCV-RNA prevalence

**Fig 2:**
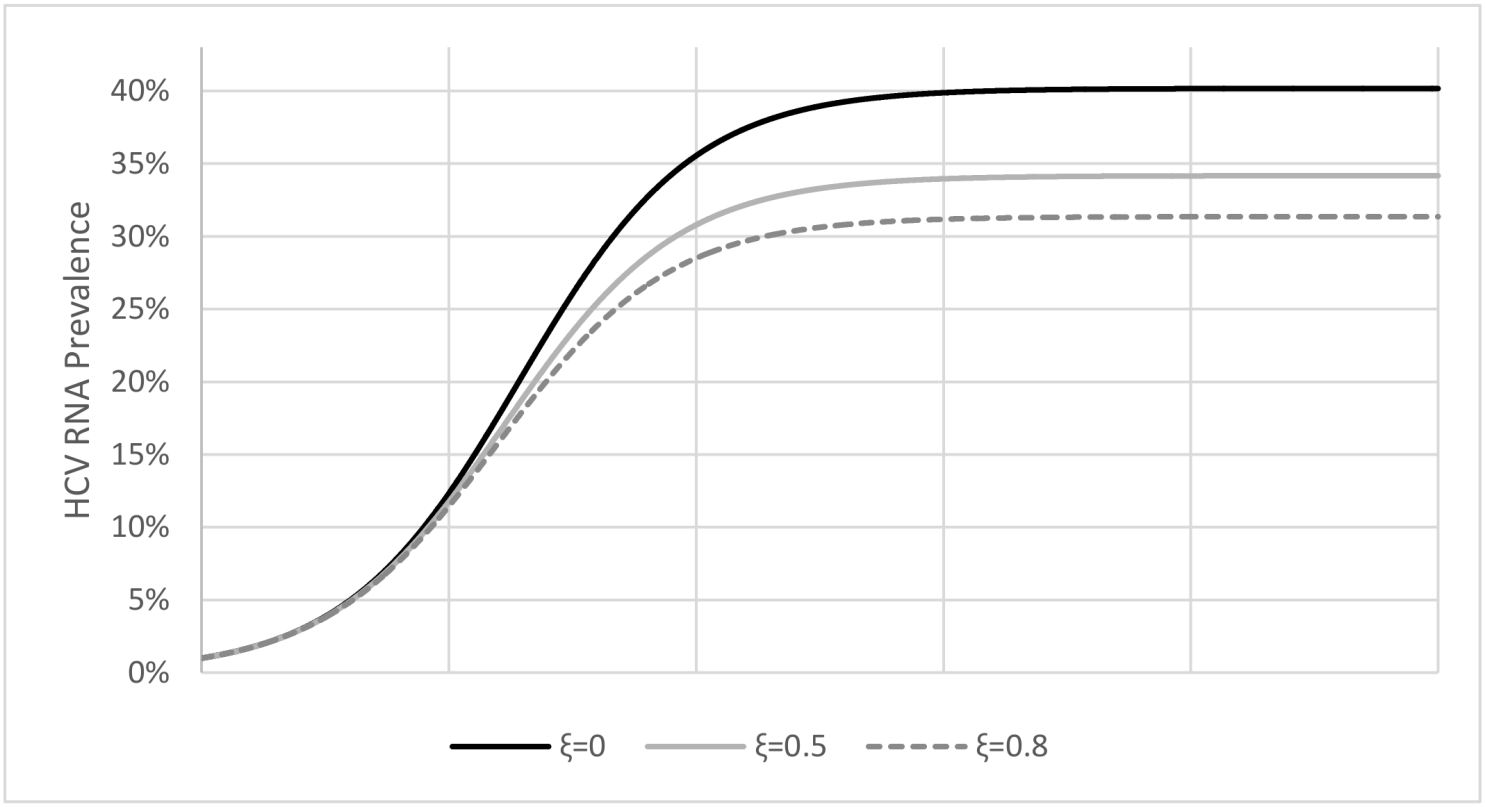
The effect of immunity (parameter ξ) on HCV-RNA prevalence. With no immunity (i.e., ξ = 0) HCV-RNA prevalence reaches steady state at 40% (solid black line). When 50% or 80% of cases result in immunity, HCV-RNA prevalence reaches steady state at 34% (solid gray line) or 31% (dashed gray line), respectively. All other model parameters were set as shown in Table 1 and π = 0.192. The simulations were initiated with one HCV-infected individual until it reached steady state with no treatment scale-up. On the x-axis each rectangle represents 50 years.

When we analyzed the impact of a high rate of immunity resulting from spontaneous HCV clearance after exposure (parameter ξ = 80%), while keeping all other parameters intact, the model showed lower HCV prevalence compared to moderate (ξ = 50%) or no immunity (ξ = 0) as assumed in Martin et al. [17] (Fig 2). However, reproducing an empirical HCV-RNA prevalence (defined as H_RNA_P = (C_1_+C_2_)/N; where N is the total baseline (or pretreatment) PWID population (i.e., *X*+*C*_1_+*C*_2_+*Z*) in a given population), an increased level of immunity may lead to counter intuitive results. For example, the assumption of a high spontaneously acquired immunity rate (ξ = 0.8) implies that the rate of infection (π) must be higher in order to sustain the same prevalence. S1 Table shows the infection rates needed to sustain baseline prevalence of 20% to 60% with varying acquired immunity rates (ξ is varied between 0 and 0.8). If acquired immunity of ξ = 0.8 is assumed, an infection rate of 0.494 is required to obtain 60% prevalence (right hand column). This contrasts with an infection rate of only 0.287 to obtain 60% prevalence when no acquired immunity is assumed (ξ = 0).

### The effect of short duration of therapy and high SVR rates with treatment scale-up of 10 infections per 1,000 PWID on HCV-RNA prevalence

**Fig 3:**
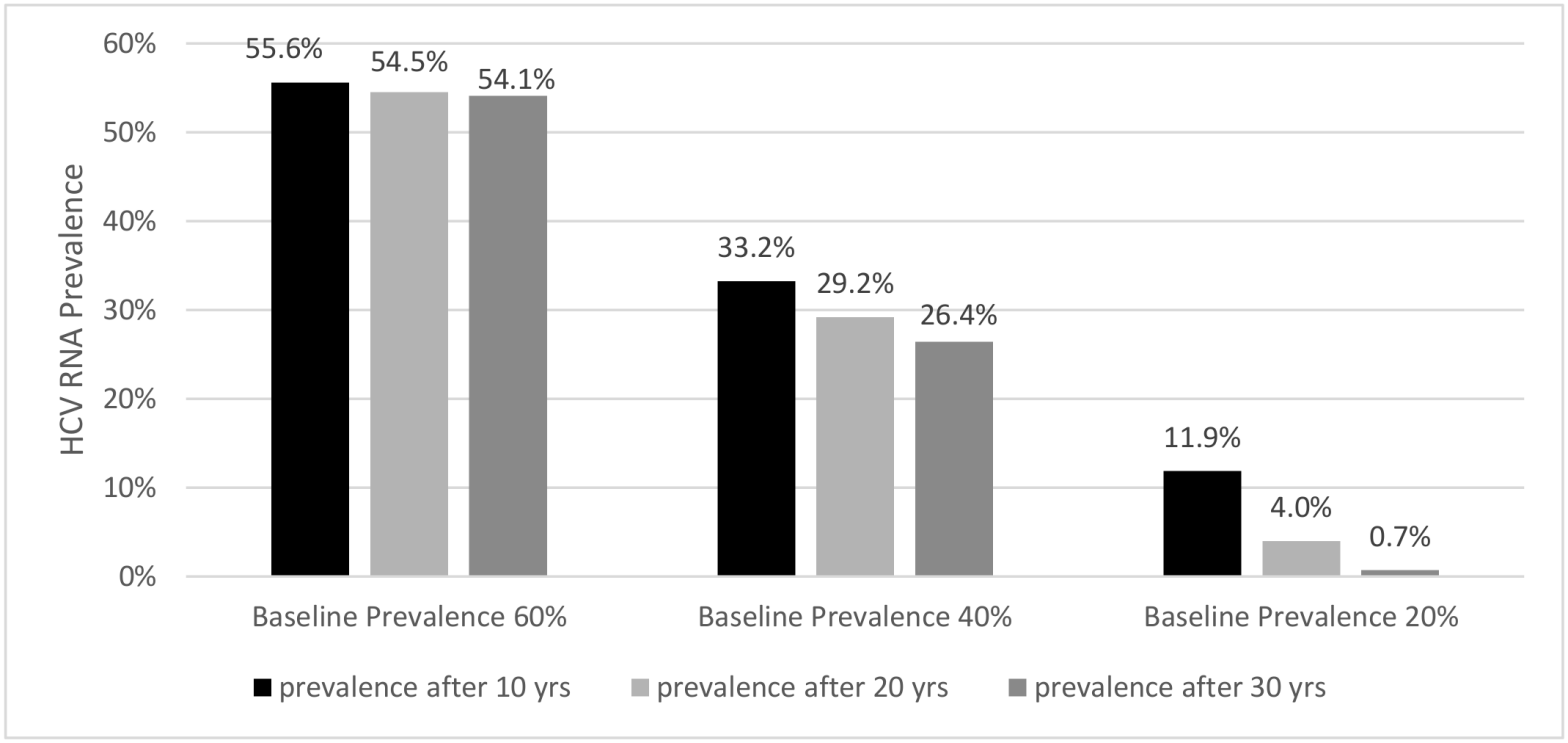
Effect of baseline HCV-RNA prevalence and differential duration of scale-up campaign (10, 20 and 30 years). Based on scale-up of 10 infection per 1000 PWID with a high proportion of cured infection (SVR 90%), a 12-week treatment duration, and acquired immunity (ξ = 0.8).

The effect of high immunity (ξ = 0.8), shorter treatment durations, and high SVR rate on HCV-RNA prevalence were explored. We assume that PWID who are cured by treatment do not develop acquired immunity (i.e., σ = 1 in Table 1; see also S5 Table). Shorter treatment durations of 6 versus 12 weeks have an insignificant effect on H_RNA_P reduction, with differences in prevalence < 1% (not shown). Shorter durations might increase compliance although this is not considered in the model. The model predicts that high SVR rates (90%) and short duration of treatment (6 or 12 weeks) will not produce a benefit with a fixed treatment scale-up of 10 infections per 1,000 in PWID populations with high (>40%) baseline H_RNA_P (Fig 3). However, among populations with a low baseline H_RNA_P (~20%), treatment scale-up of 10 infections per 1,000 PWID will effectively reduce HCV-RNA prevalence (Fig 3). Assuming no immunity (ξ = 0) as in Martin et al, the HCV RNA prevalence reduction over 30 years with immunity (ξ = 0.8) is 1.5%, 6.8%, and 11.2% and without immunity (ξ = 0) the reduction is 2.5%, 8.9%, and 11.1% for baseline prevalence 60%, 40%, and 20%, respectively (compare Fig 3 with S1 Fig).

### HCV Epidemiology in the Chicago PWID population and estimated incidence of infection

Table 2 provides the estimates for PWID population size, median age, and prevalence of HCV-Ab and HCV-RNA in Chicago [20, 25]. In addition to characterizing the overall PWID population, we report on two sub-groups, i.e., those attending HR program and young (<30 years old) PWID, that show distinct health profiles that are supported by epidemiological trends [21, 37]. For the total Chicago PWID population with RNA prevalence of 47%, modeling predicts an incidence rate of HCV infection of 11.2 (or 7.4 without immunity) cases per 100 person-years (PY). For the HR and young PWID subpopulations (RNA prevalences of 30% and 10%, respectively), incidence rates of 5.4 (or 3.4 without immunity) cases per 100 PY and 1.3 (or 0.9 without immunity) cases per 100 PY, respectively, were predicted.

**Table 2:** Prevalence and treatment scale-up estimates.

Population	Approx. Pop. Size (n)	Median Age (IQR)	HCV-Ab+ prevalence, H_AB_P (%)	HCV RNA+ prevalence H_RNA_P (%)	Infection rate (π)	Scale-up per 1000 PWID to halve the HCV RNA prevalence in 10 years [min-max]*	($M) over 10 years [min-max]**
**ALL**	32,000^@^	44 (35–52)	59^A^	47^C^	.289	35 [31–48]	560 [496–768]
**Harm Reduction (HR)**	22,000	45 (35–52)	38^A^	30^C^	.187	19 [18–24]	209 [198–264]
Young PWID (HR and non-HR)*	11,000	27 (24–28)	14^B^	10^D^	.15	5 [5–7]	28 [28–39]

HR, PWID in harm reduction programs;

^@^, based on the population estimate from Tempalski et al [20];

IQR, interquartile range;

^A^, data from the CDC-sponsored National HIV Behavioral Surveillance System (NHBS09) of 2009;

^B^ data from the Third Collaborative Injection Drug Users (CIDUS III) study;

^C^ was calculated using H_RNA_P~H_AB_P *(1- ξδ) where ξ = 0.8 and δ = 0.26;

^D^, based on empirical data of HCV-RNA and HCV-Ab measurements (see Methods) which translated into δ = 0.34 [25]; M, million;

** based on sensitivity analysis (S2–S6 Tables).

*Note that sub-populations are overlapping (see Discussion).

### Predicting the effect of treatment scale-up in metropolitan Chicago

**Fig 4:**
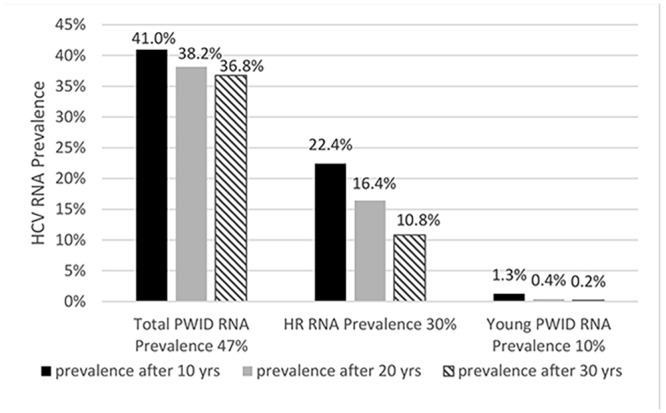
Resulting H_RNA_P after 10, 20 and 30 years of treating 10 infections per 1000 PWID with 90% SVR rate, 12-week treatment duration and acquired immunity (ξ = 0.8). Predicted effect from Chicago’s overall baseline H_RNA_P (47%), Chicago’s harm reduction (HR) attending PWID (30%) and Chicago’s subpopulation with low baseline H_RNA_P (10%).

As shown in Fig 4, the model predicts that treating 10 infected individuals per 1,000 PWID for 12 weeks with 90% SVR rate and acquired immunity rate, ξ = 0.8 does not yield a significant reduction in H_RNA_P for the total PWID population with an HCV-RNA prevalence of 47% after 10, 20, and 30 years of treatment (Fig 4). However, among sub-populations with a low baseline H_RNA_P (such as PWID attending HR programs with baseline H_RNA_P = 30%, or young PWID with baseline H_RNA_P = 10%; Table 2), treatment scale-up of 10 individuals per 1,000 PWID will effectively reduce HCV-RNA prevalence (Fig 4).

The treatment scale-up needed to reduce the baseline H_RNA_P by one-half was calculated considering an acquired immunity rate ξ = 0.8, a 90% SVR rate, and a 12 week treatment duration (Table 2). We first estimated the H_RNA_P of each empirical H_AB_P as described in Table 2. In all cases except for young PWID, we assumed that δ = 0.26 as in Martin et al. [17]. In a previous study of a young PWID population [25], we found that 34% of HCV-Ab positive young PWID cleared the virus. Using δ = 0.34 for the young PWID, if HCV-Ab positive prevalence, H_AB_P = 14%, then only ~10% will be chronically infected (HCV-RNA positive; Table 2). Considering these baseline H_RNA_P estimates, the model indicates that the following scale-up is required to reduce the baseline HCV-RNA prevalence by one-half within 10 years of initiating treatment (Table 2): 35/1000 PWID among the total Chicago PWID population, 19/1000 PWID in harm reduction (HR) programs, and 5/1000 young PWID.

### Sensitivity Analysis

For the sensitivity analysis, we focused on the effect of the parameters on the scale-up needed to reduce the prevalence by one-half in 10 years. Overall, when the prevalence is low (H_RNA_P <40%), as among young PWID sub-populations, we found low sensitivity to parameters in a one-way analysis (S2–S6 Tables). Additional analyses showed that the higher the prevalence, the greater the disparity in treatment scale-up between the extreme low and high values of the parameters tested. The two most influential parameters are the (i) PWID attrition rate by death or cessation of injection behavior (parameter μ) (S2 Table) and (ii) the average proportion of cured PWID (i.e., SVR; parameter α) (S6 Table). The outcome is much less influenced by the rate of acquired immunity, either following treatment (S5 Table) or through self-clearance (S4 Table).

### Cost-Impact Analysis

We estimated the cost benefit of treatment for the 32,000 total PWID in Chicago as well as the sub-populations delineated in Table 2. The listed prices of the recently approved DAAs in the U.S. are $84,000 (sofosbuvir), $66,000 (simeprevir), $94,500 (ledipasvir-sofosbuvir) and $83,319 (ombitasvir/paritaprevir/ritonavir; dasabuvir) for a 12-week course of therapy [10]. The DAAs are being discounted by 40–50% and therefore we chose a conservative cost of $50,000 per person. A treatment scale-up of 35 infections per 1,000 PWID annually among the total PWID residing in metropolitan Chicago would cost an estimated $50-$77 million per year of treatment (Table 2). For PWID sub-populations such as HR and young persons, a treatment scale-up per year of treatment of 19 and 5 infections per 1,000 PWID would cost approximately $20-$26 and $3-$4 million, respectively. Assuming no immunity as in Martin et al [17] (compared to with immunity ξ = 0.8), the cost to reduce the number of infections per 1000 by one half in 10 years would be $3.2M per year for the total Chicago PWID population, $1.1M annually for HR, and $0.5M a year for young PWID, (S4 Table).

## Discussion

The largest reservoir of on-going HCV transmission occurs among PWID, and HCV incidence among the young is increasing [31, 35, 38–42]. While successful treatment of HCV in PWID could have a substantial impact on disease transmission, less than 1% of HCV-infected PWID are treated annually in the U.S. [6, 7]. A major barrier to treatment of this population could be alleviated with IFN-free all-oral DAAs therapeutic regimens. However, the issue of cost will need to be considered. The current study examines HCV treatment scale-up and associated projected costs in Chicago, which is a large U.S. metropolitan area with substantive epidemiological data on the PWID population. The model suggests that to reduce the HCV-RNA prevalence (currently ~47%) of the entire PWID population in Chicago by half over a 10 year period would require treatment scale-up of 35 (per 1,000) with a staggering estimated cost for the DAAs alone of $50-$77 million per year. In PWID sub-populations with low HCV-RNA prevalence, e.g., 10% among young (<30 yrs old) PWID, a treatment scale-up of 5 (per 1,000) would cost $3-$4 million per year (for DAAs only) in order to decrease the HCV-RNA prevalence by one half over 10 years of treatment. The latter prediction suggests that treatment scale-up among the young PWID populations is feasible and could achieve desirable outcomes.

In this study, we assume that 12 week DAA cost alone after 40%-50% discounting and without other associated medical costs (e.g., monitoring HCV-RNA for response), is $50,000 per patient. Hill et al. [43] projected that, within the next 15 years, large-scale manufacture of DAAs could reduce the manufacturing cost for a 12 week course to as low as $100–$250 per person. Until the cost of DAAs decreases substantially in the U.S., identifying targeted strategies for treatment scale-up is imperative to reduce HCV transmission and prevalence.

Our sensitivity analysis indicated that one of the most influential parameters on cost is the average cure (or SVR) rate of DAA treatment. While high SVR rates (~90%) may be achieved in individuals with high levels of adherence (i.e., near optimal adherence among persons enrolled in clinical trials), the SVR rate might be much lower among some PWID due to lower adherence rates. For example, our analysis suggests that therapy with a low SVR rate (70%) would be about 1.3-fold more expensive than the cost of treatment with a high SVR rate (90%) (S6 Table). Although some parameters have a moderate effect on HCV-RNA prevalence reduction (e.g., the presence or absence of acquired immunity), it is important to note that small changes in treatment scale-up may lead to a significant increase in cost when treatment is scaled up from 1,000 PWID to the population level (e.g., S4 Table). Notably, a recent meta-analysis of IFN-based therapies in PWID indicated similar SVR rates to those obtained in the general population and that more than 80% of PWID successfully completed treatment [44, 45]. Given that these studies used IFN-based therapies and that all-oral DAAs are significantly less toxic, even higher completion rates are expected among PWID in future scale-up trials.

In agreement with Martin et al. [17], our findings suggest that in areas with very high prevalence it would be prohibitively expensive to mount an effective campaign to treat all HCV-infected PWID. Recent epidemiological trends support a focus on young PWID as they represent the source of on-going HCV transmission due to the preponderance of evidence supporting that younger age is associated with higher risk injection behaviors among PWID [24, 46–49]. As such, even though HCV prevalence has steadily declined among older PWID [50], high incidence and recent outbreaks of HCV infection among younger PWID continues [31, 35, 38–42], partly attributable to higher levels of risky injection behaviors among young PWID compared to their older counterparts [22, 51, 52]. Recent data on young Chicago PWID (Boodram et al. unpublished) show that young PWID (median age = 26) are likely to share with slightly older PWID (median = 30). Given the higher HCV prevalence among older PWID, young PWID are at high risk for HCV infection and reinfection. Reducing the pool of infections among this group will be imperative to reducing new HCV infections over time and could have a more substantive long-term impact on the HCV epidemic in the U.S.

It has been shown that spontaneous clearance of primary HCV infections results in a form of immunity that controls secondary infections and leads to rapid clearance in the majority of cases [12, 30]. Such immunity to chronic infection is expected to have an impact on the spread of HCV infection among the PWID population. Indeed, our model shows that higher infection rates need to be assumed in order to achieve equivalent prevalence in the starting population. This impact of immunity on treatment scale-up requires further modeling and analysis particularly given the data showing that individuals treated during the acute phase of an infection develop functional CD4+ and CD8+ responses [14, 15]. This could result in larger numbers of immune subjects within a population as treatment is undertaken and could have a greater overall impact in younger PWID as the probability of treating individuals that are in the acute phase of infection would also increase. Although a vaccine against HCV is still some way off [53], a future combination of vaccination and DAA treatment of PWID would add a new dimension to the spread and subsequent control of the disease which merits further modeling analyses.

### Limitations

While our modeling approach (termed a *compartmental model*) is the most frequently used class of models for studying HCV infection and treatment among PWID, it has well-known limitations [16]. Compartmental models such as the one used in this study assume that the population in each HCV infection state is homogeneous and that the population is totally mixed. Although several parameters were obtained from observational studies (Table 1), they are not specific to the Chicago area and may not be relevant to all PWID subpopulations. In addition, in this study each sub-population (i.e., HR attending PWID and young PWID) was modeled separately, based on its empirical baseline HCV prevalence, adjusting the HCV infection rate accordingly (π; see S1 Table). However, in reality sub-populations are overlapping, therefore treating one sub-population would have an effect on other sub-populations, which the current model is not designed to predict. For example, treatment of young PWID attending HR programs is expected to have a larger impact than what our model suggested for treating all young PWID. Since not all PWID have contacts to other PWID, additional in-depth modeling analysis of antiviral scale-up in Chicago would require a more detailed representation of the network of contacts [54] within and across populations such as used in agent-based modeling (ABM) or individual-based modeling (reviewed in [16]). In addition, ABM has the potential to simulate changes in drug injection behavior, PWID demographics and population size to address variability in characteristics that will likely change over a 10 to 20 year treatment period.

### Conclusion

Our study is the first to report on DAA-treatment scale-up and associated cost in a large U.S. metropolitan area. There has been consistently low uptake of interferon-based treatment among PWID. The FDA-approval of IFN-free all oral therapy with DAAs provides the opportunity for a treatment-as-prevention approach in the high risk PWID population. Our model indicates the cost of DAA therapy for all HCV infected PWID in the Chicago metropolitan area may be prohibitive. However, targeting therapy to specific groups such as young PWID could have a major impact at a more practical cost. The model presented here provides a starting point for cost-effective analysis and more comprehensive modeling and field data efforts.

## Supporting Information

S1 Eq Model equations. Variables and parameters are described in Methods, Fig 1 and Table 1 in main text.
(DOCX)

S1 Fig Effect of baseline HCV-RNA prevalence and differential duration of scale-up campaign (10, 20 and 30 years). Based on scale-up of 10 infection per 1000 PWID with a high proportion of cured infection (SVR 90%), a 12-week treatment duration, and no acquired immunity (ξ = 0).
(PDF)

S1 Table The infection rate needed to sustain baseline prevalences of 20%, 30%, 40%, 50%, and 60% with varying ξ values. Based on scale-up of 10 infections per 1000 PWID, 12 week treatment duration, and δ = 0.26.
(PDF)

S2 Table One way sensitivity analysis conducted on the average new injector rate (θ) and average PWID leaving rate by cessation or death (μ) and the effects on scale-up treatment needed to reduce the baseline RNA prevalence by ½ in 10 years. We maintain θ = 1000*μ to ensure constant N = 1000.
(PDF)

S3 Table One way sensitivity analysis conducted on the average proportion of infections that spontaneously clear the infection (delta = δ) and the effects on scale-up treatment needed to reduce the baseline RNA prevalence by ½ in 10 years. (PDF)

S4 Table One way sensitivity analysis conducted on average proportion of spontaneously cleared infections resulting in immunity (ξ) and the effects on scale-up treatment needed to reduce the baseline RNA prevalence by ½ in 10 years. (PDF)

S5 Table One way sensitivity analysis conducted on average proportion of cured infections, due to treatment, resulting in immunity (1-σ) and the effects on scale-up treatment needed to reduce the baseline RNA prevalence by ½ in 10 years. (PDF)

S6 Table One way sensitivity analysis conducted on average proportion of cured infections with sustained viral response (SVR) (α) and the effects on treatment scale-up needed to reduce the baseline RNA prevalence by ½ in 10 years. (PDF)
