# Supplementary material for: Mathematical Modeling of Hepatitis C Prevalence Reduction with Antiviral Treatment Scale-Up in Persons Who Inject Drugs in Metropolitan Chicago
Source: PLoS One. 2015 Aug 21;10(8):e0135901. doi: 10.1371/journal.pone.0135901 (PMC4546683; doi:10.1371/journal.pone.0135901)
Supplement: S1 Eq — Variables and parameters are described in Methods, Fig 1 and Table 1 in main text. (DOCX) [file pone.0135901.s001.docx]

**Supporting information**

**S1 Eq. Model equations**. Variables and parameters are described in Methods, Fig. 1 and Table 1 in main text. When C_1_ < Φ (i.e., fewer HCV-infected individuals, C1, than in treatment scale-up, Φ) we set Φ=C_1_ to maintain positivity of the equations.

$$\begin{matrix} \frac{dX}{d\tau}=-\pi\left( 1-\delta+\delta\xi\right)\frac{C_{1}+C_{2}}{N}X+\omega\alpha\sigma Tr+\theta-\mu X \\ \frac{dC_{1}}{d\tau}=\pi\left( 1-\delta\right)\frac{C_{1}+C_{2}}{N}X-\Phi-\mu C_{1} \\ \frac{dTr}{d\tau}=\Phi-\omega Tr-\mu Tr \\ \frac{dZ}{d\tau}=\pi\delta\xi\frac{C_{1}+C_{2}}{N}X+\omega\alpha\left( 1-\sigma\right)Tr-\mu Z \\ \frac{dC_{2}}{d\tau}=\omega\left( 1-\alpha\right)Tr-\mu C_{2} \end{matrix}$$
