# Supplementary material for: Mathematical Modeling of Hepatitis C Prevalence Reduction with Antiviral Treatment Scale-Up in Persons Who Inject Drugs in Metropolitan Chicago
Source: PLoS One. 2015 Aug 21;10(8):e0135901. doi: 10.1371/journal.pone.0135901 (PMC4546683; doi:10.1371/journal.pone.0135901)

Supporting information

S1 Fig. Effect of baseline HCV-RNA prevalence and differential duration of scale-up campaign (10, 20 and 30 years). Based on scale-up of 10 infection per 1000 PWID with a high proportion of cured infection (SVR 90%), a 12-week treatment duration, and no acquired immunity (ξ=0)

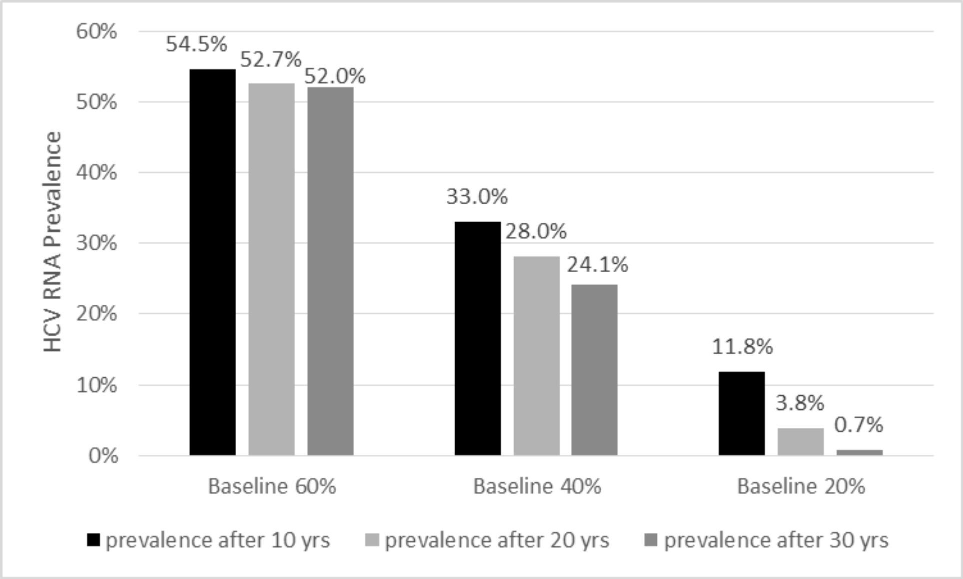

Supplement: S1 Fig — Based on scale-up of 10 infection per 1000 PWID with a high proportion of cured infection (SVR 90%), a 12-week treatment duration, and no acquired immunity (ξ = 0). (PDF) [file pone.0135901.s002.pdf]
