# Supplementary material for: Mathematical Modeling of Hepatitis C Prevalence Reduction with Antiviral Treatment Scale-Up in Persons Who Inject Drugs in Metropolitan Chicago
Source: PLoS One. 2015 Aug 21;10(8):e0135901. doi: 10.1371/journal.pone.0135901 (PMC4546683; doi:10.1371/journal.pone.0135901)
Supplement: S1 Table — Based on scale-up of 10 infections per 1000 PWID, 12 week treatment duration, and δ = 0.26. (PDF) [file pone.0135901.s003.pdf]

Supporting information

S1 Table. The infection rate needed to sustain baseline prevalences of 20%, 30%, 40%, 50%, and 60% with varying ξ values. Based on scale-up of 10 infections per 1000 PWID, 12 week treatment duration, and δ= 0.26.

ξ	Infection rate (Prevalence 20%)	Infection rate (Prevalence 30%)	Infection rate (Prevalence 40%)	Infection rate (Prevalence 50%)	Infection rate (Prevalence 60%)
0.8	.155	.187	.236	.319	.494
0.7	.153	.184	.229	.305	.454
0.6	.152	.181	.223	.291	.419
0.5	.151	.178	.217	.279	.391
0.0	.144	.164	.192	.23	.287
