# Supplementary material for: Mathematical Modeling of Hepatitis C Prevalence Reduction with Antiviral Treatment Scale-Up in Persons Who Inject Drugs in Metropolitan Chicago
Source: PLoS One. 2015 Aug 21;10(8):e0135901. doi: 10.1371/journal.pone.0135901 (PMC4546683; doi:10.1371/journal.pone.0135901)
Supplement: S2 Table — We maintain θ = 1000*μ to ensure constant N = 1000. (PDF) [file pone.0135901.s004.pdf]

Supporting information

S2 Table. One way sensitivity analysis conducted on the average new injector rate (theta=θ) and average PWID leaving rate by cessation or death (mu=μ) and the effects on scale-up treatment needed to reduce the baseline RNA prevalence by ½ in 10 years. We maintain θ =1000*μ to ensure constant N=1000.

		Extreme low			Extreme high			Cost per PWID population per year
Population	RNA+ prevalence (%)	Theta/μ	Infection rate (π)	Scale -up	Theta/μ	Infection rate (π)	Scale -up	$M
ALL	47	50/.05	.17	31	200/0.2	.68	48	49.6-76.8
HR	30		.11	18		.439	22	19.8-24.2
Young PWID	10		.088	5		.353	6	2.8-3.3
