# Supplementary material for: Mathematical Modeling of Hepatitis C Prevalence Reduction with Antiviral Treatment Scale-Up in Persons Who Inject Drugs in Metropolitan Chicago
Source: PLoS One. 2015 Aug 21;10(8):e0135901. doi: 10.1371/journal.pone.0135901 (PMC4546683; doi:10.1371/journal.pone.0135901)
Supplement: S3 Table — (PDF) [file pone.0135901.s005.pdf]

## Supporting information

**S3 Table. One way sensitivity analysis conducted on the average proportion of infections that spontaneously clear the infection (delta= δ) and the effects on scale-up treatment needed to reduce the baseline RNA prevalence by ½ in 10 years.**

| | | Extreme low | | | Extreme high | | | Cost per PWID population per year |
|---|---|---|---|---|---|---|---|---|
| Population | RNA+ prevalence (%) | δ | Infection rate (π) | Scale-up | δ | Infection rate (π) | Scale-up | $M |
| ALL | 47 | .22 | .258 | 34 | .34 | .383 | 36 | 54.4-57.6 |
| HR | 30 | | .172 | 18 | | .223 | 19 | 19.8-20.9 |
| Young PWID | 10 | | .1243 | 5 | | .15 | 5 | 2.8 |
