# Supplementary material for: Mathematical Modeling of Hepatitis C Prevalence Reduction with Antiviral Treatment Scale-Up in Persons Who Inject Drugs in Metropolitan Chicago
Source: PLoS One. 2015 Aug 21;10(8):e0135901. doi: 10.1371/journal.pone.0135901 (PMC4546683; doi:10.1371/journal.pone.0135901)
Supplement: S4 Table — (PDF) [file pone.0135901.s006.pdf]

Supporting information

S4 Table. One way sensitivity analysis conducted on average proportion of spontaneously cleared infections resulting in immunity (ξ) and the effects on scale-up treatment needed to reduce the baseline RNA prevalence by ½ in 10 years.

		Extreme low			Extreme high			Cost per PWID population per year
Population	RNA+ prevalence (%)	(ξ)	Infection rate (π)	Scale-up	(ξ)	Infection rate (π)	Scale-up	$M
ALL	47	0	.217	33	0.8	.289	35	52.8-56
HR	30		.164	18		.187	19	19.8-20.9
Young PWID	10		.144	5		.15	6	2.8-3.3
