# Supplementary material for: Mathematical Modeling of Hepatitis C Prevalence Reduction with Antiviral Treatment Scale-Up in Persons Who Inject Drugs in Metropolitan Chicago
Source: PLoS One. 2015 Aug 21;10(8):e0135901. doi: 10.1371/journal.pone.0135901 (PMC4546683; doi:10.1371/journal.pone.0135901)
Supplement: S5 Table — (PDF) [file pone.0135901.s007.pdf]

Supporting information

S5 Table. One way sensitivity analysis conducted on average proportion of cured infections, due to treatment, resulting in immunity (1-σ) and the effects on scale-up treatment needed to reduce the baseline RNA prevalence by ½ in 10 years.

		Extreme low immunity rate			Extreme high immunity rate			Cost per PWID population per year
Population	RNA+ prevalence (%)	(1-σ=0)	Infection rate (π)	Scale-up	(1 -σ=.25)	Infection rate (π)	Scale-up	$M
ALL	47	σ=1	.289	35	σ=0.75	.289	33	52.8-56
HR	30		.187	19		.187	18	19.8-20.9
Young PWID	10		.15	6		.15	6	3.3
