# Supplementary material for: Mathematical Modeling of Hepatitis C Prevalence Reduction with Antiviral Treatment Scale-Up in Persons Who Inject Drugs in Metropolitan Chicago
Source: PLoS One. 2015 Aug 21;10(8):e0135901. doi: 10.1371/journal.pone.0135901 (PMC4546683; doi:10.1371/journal.pone.0135901)
Supplement: S6 Table — (PDF) [file pone.0135901.s008.pdf]

Supporting information

S6 Table. One way sensitivity analysis conducted on average proportion of cured infections with sustained viral response (SVR) (α) and the effects on treatment scale-up needed to reduce the baseline RNA prevalence by ½ in 10 years.

		Extreme low			Extreme high			Cost per PWID population per year
Population	RNA+ prevalence (%)	Alpha (α)	Infection rate (π)	Scale-up	Alpha (α)	Infection rate (π)	Scale-up	$M
ALL	47[C]	70%	.289	44	90%	.289	35	56-70.4
HR	30[C]		.187	24		.187	19	20.9-26.4
Young PWID	10[D]		.15	7		.15	6	3.3-3.9
